# Establishing the health and wellbeing needs of mining host community in Brazil, Chile and Peru: a mixed-method approach to identify priority areas for action to help communities meet their SDG3 targets

**DOI:** 10.1186/s12889-023-17017-4

**Published:** 2023-11-10

**Authors:** Brian Rice, Ana Maria Buller, Delia Boccia, Cesar Bazan, Rafael Miranda, Ximena Cea, Rodrigo Laro, Miguel Fontes, Rosalie Hayes, Mariken de Wit, Daniel J. Carter, Alexandra Plowright, Matthew Chadwick, Mitzy Gafos

**Affiliations:** 1 London School of Hygiene & Tropical Medicine, 15-17 Tavistock Place, London, WC1H 9SH UK; 2 Sheffield Centre for Health and Related Research (SCHARR), School of Medicine and Population Health, University of Sheffield, Sheffield, UK; 3 Innovation for Development (I4D), Lima, Peru; 4 Universidad Continental del Peru, Junín, Peru; 5 Universidad Andrés Bello, Santiago, Chile; 6 John Snow Brasil, Brasilia, Brazil; 7 Anglo American Plc, London, UK

**Keywords:** SDG, Latin America, Mixed-methods, Health, Wellbeing, Peru, Brazil, Chile

## Abstract

**Background:**

The global mining industry is an important partner in advancing the 2030 Agenda for Sustainable Development. In 2018, Anglo American plc published their Sustainable Mining Plan, containing a goal for improving health and wellbeing aligned with the Sustainable Development Goal 3 (SDG3) targets. Having formed an independent multidisciplinary research consortium, we designed and implemented a mixed-methods approach to attain a deeper understanding of SDG3 priorities within the local context of communities hosting Anglo American mining operations located in Latin America.

**Methods:**

In 2019, within the host communities of three mining operations in Chile, three in Brazil, and one in Peru, we conducted a qualitative study which included stakeholder workshops and key informant interviews. We also quantitatively appraised existing health data. Findings emerging from the qualitative and quantitative assessments were compared to identify health and wellbeing priority areas for action relevant to each community.

**Results:**

Across the three countries, 120 people took part in workshops and 35 in interviews. In these workshops and interviews, non-communicable diseases (SDG3.4), harmful alcohol consumption (SDG3.5), and pollution, particularly air pollution (SDG3.9), were consistently identified as areas for priority action. There were similarities in the reporting of individual, interpersonal, community, societal, and structural factors underlying these priority areas across the different communities**.** The availability of quantitative data was generally good at the state level, becoming increasing sparse as we focused on smaller geographies. The priorities identified in the quantitative assessments generally aligned with those highlighted in the qualitative data.

**Conclusions:**

We highlight the importance of engaging with local populations to understand and address health needs. To address the priorities identified, intervention packages tailored to the specific needs of host communities, that tackle associated upstream societal level factors, are required. To facilitate this, appropriate monitoring systems and epidemiological investigations should be implemented to better understand the local context and quantify health issues. In the host communities, it is essential for the mining sector to be a key health partner in promoting integrated programmes that contribute to achieving the priority objectives and targets aligned with the SDG3 agenda.

## Background

Established in 2015 by the United Nations General Assembly, the Sustainable Development Goals (SDGs) are seventeen interlinked global goals designed to achieve, by 2030, a better and more sustainable future for all [1]. To achieve a sustainable future, the SDGs focus on promoting healthy lives, eliminating poverty and inequalities, protecting the planet from degradation, and ensuring all people enjoy peace and prosperity.

Although the potential negative health and environmental costs of industry are well documented [2, 3], so too is the potential for a mutually reinforcing relationship between sustainable social and industrial development driven by, among other things, employment creation and poverty eradication [4]. Sustainable Development Goals 9 and 17 focus on the importance of strengthening and revitalising global multisector partnerships and the role that resilient infrastructure and innovation, and inclusive and sustainable industrial development, has in achieving a sustainable future [5]. An industrial sector that has come under particular scrutiny in relation to its potential for driving economic development in settings that are often remote and less developed is large-scale extraction projects. In 2016, members of the United Nations Development Programme and World Economic Forum identified the global mining industry as having an unprecedented opportunity to advance the 2030 Agenda for Sustainable Development [6]. Responding to the SDGs presents the mining industry with an opportunity to mitigate some of the adverse health impacts associated with their activities. Health impacts may either be direct due to toxic work and environmental exposures, or indirect via mechanisms such as water insecurity and stress-related ailments [7].

In 2018, acknowledging the role they could play in redressing issues that are prominent in terms of negative health outcomes in countries of operation, and in advancing progress against the SDGs, Anglo American plc published their Sustainable Mining Plan [8]. The health and wellbeing goal within this plan aligns with the SDG3 targets. In 2018, the London School of Hygiene and Tropical Medicine (LSHTM) was commissioned by Anglo American and the Anglo American Foundation to form an independent academic consortium to identify community-specific priority areas for action under SDG3, and direct where interventions should be targeted.

In early 2019, a group of multidisciplinary researchers at LSHTM established the Sustainable Development Goals Health and Wellbeing consortium. Having identified local research partners, we designed and applied a mixed-methods approach to assess health and wellbeing priorities in the host communities of the seven Anglo American mining operations located in three countries (Brazil, Chile, and Peru) within the Latin America and Caribbean (LAC) region.

## Methods

**Fig. 1:**
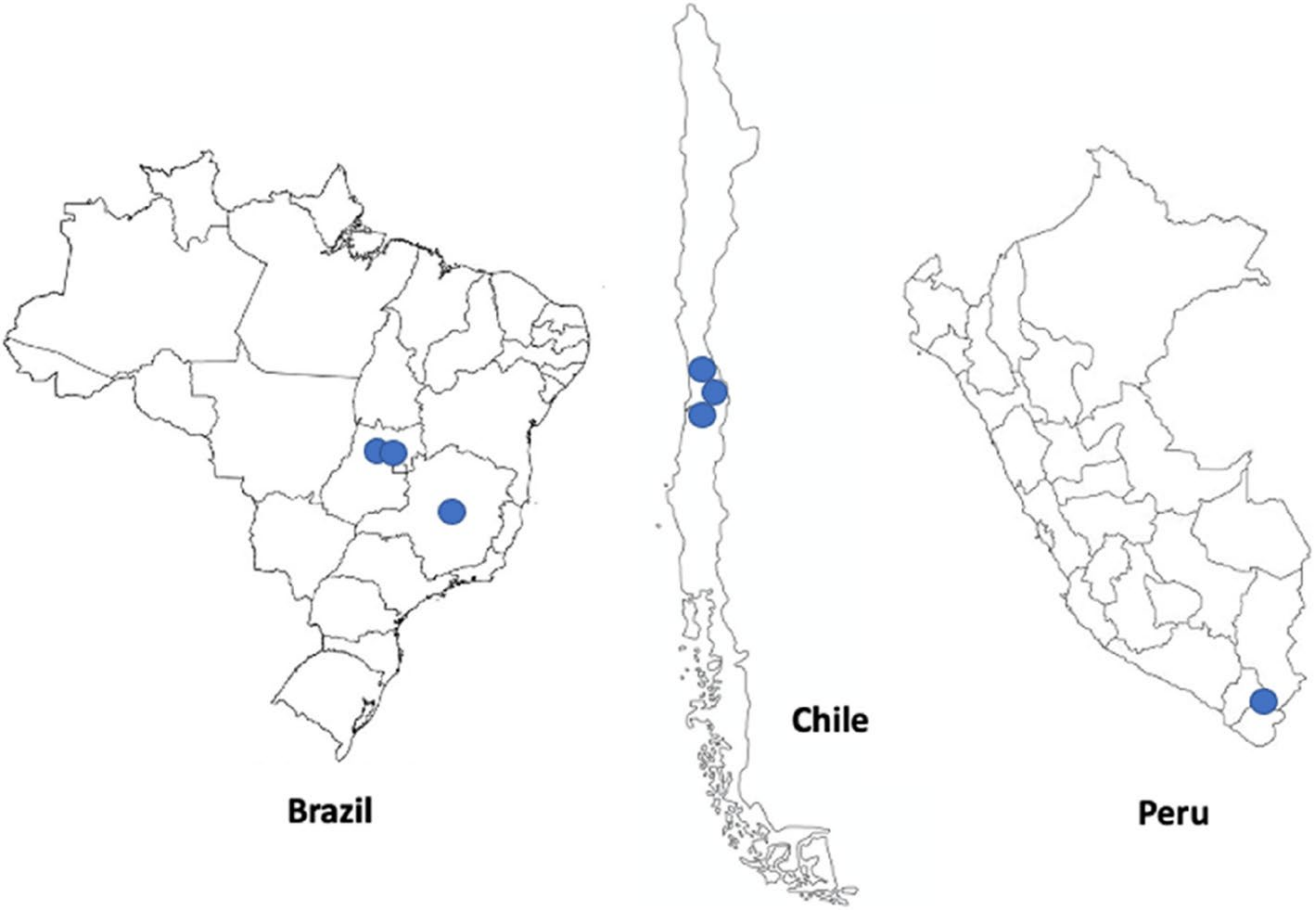
Mine locations. Brazil: three mines located in the Federal States of Goiás and Minas Gerais. Chile: three mines located in the Valparaíso region and the Metropolitan Region of Santiago de Chile. Peru: one mine was located in the Mariscal Nieto province in the Moquegua region

Details of our mixed-methods research have been described elsewhere [9]. In summary, to assess health and wellbeing priorities in the host communities of three Anglo American mining operations in Chile, three in Brazil, and one in Peru (for the latter, the mine was in its set-up phase during the study period) we quantitatively appraised existing publicly available health data and conducted qualitative research with stakeholders from the host communities. These communities were identified by the mine teams and based on proximity to the mining operations irrespective of an employment relationship with Anglo American. In Brazil, the mines were located in the Federal States of Goiás and Minas Gerais, whilst in Chile the mines were located in the Valparaíso region and the Metropolitan Region of Santiago de Chile (see Fig. 1). In Peru, the mine was located in the Mariscal Nieto province in the Moquegua region. Due to challenges in accessing the highland region of Mariscal Nieto where the Anglo-American mine was being established, we based our study in Moquegua City. Given the region’s connections to multiple mining operations, we designated the entire region as the host community. Representatives from the highland communities were among the participants.

To successfully implement the assessments, it was important to understand the local socio-cultural context and facilitate communication in the language of participants. Partners with research already embedded in the countries were identified to form a Latin America research team within the consortium. In Peru and Chile, the partner was Innovation for Development, and in Brazil it was John Snow Brasil.

Between August and November 2019, we conducted qualitative research which included in-person stakeholder workshops and face to face key informant interviews. Study participants were recruited by mine social-performance teams, other stakeholders, and/or by the Latin America research teams. All participants were presented with a Participant Information Sheet (specific to workshops or interviews), and written informed consent was sought. A workshop guide was established using a participatory action research approach [10], and semi-structured key informant interview guides were developed for stakeholders (including local women’s groups, NGOs, schoolteachers, and health service providers, as well as local, regional, and national level representatives of the Ministry of Health, mine staff, and health service managers). The guides were semi-structured and covered participant’s experience and involvement in health and wellbeing improvement, key considerations for achieving SDG3 targets and gaps in the response, and suggestions for priority action. Mine staff were not invited to participate in the workshops as their presence may have influenced the extent to which stakeholders felt free to express their views. Mine staff were invited for key informant interviews.

Participants identified three or four priority areas, and their underlying factors, for action relevant to their community. Data arising from each workshop and key informant interview were analysed separately and then combined using framework analysis. To explore the interdependence between multiple determinants, using a socio-ecological model [11], participant responses were categorised to identify structural, societal, community, interpersonal and individual-level underlying factors relating to each prioritised target.

Between June and December 2019, we conducted an assessment of available quantitative health and social determinant data for each SDG3 target and indicator, in each mine location. Data sources included the SDGs United Nations Indicators website [12], governmental and non-governmental reports, and Census data. Between January to February 2020, a further non-systematic review of the scientific literature was conducted, and key experts contacted. Based on the extent to which their burden exceeded their respective target, SDG indicators were priority ranked.

An SDG3 indicator or sub-indicator was identified as a priority area when selected by multiple stakeholders in workshops and/or interviews and when the quantitative data, where available, indicated a clear burden of disease in the community above the national or the designated SDG targets. The study protocol was approved by the LSHTM research ethics committee (ref: 16,349), by UnicCEUB Research Ethics Committee in Brazil, Andrés Bello University in Chile, and San Martin de Porres University in Peru.

## Results

A total of 120 people took part in the workshops across all three countries, with 35 participating in interviews. Table 1 presents participants by country, sex and stakeholder type. In Brazil we conducted two workshops with 31 stakeholders. One workshop covered the host communities of two of the three mining operations. As a result of non-attendance, government agencies were under-represented at both workshops; to address this, government representatives were targeted for key informant interviews, accounting for two of thirteen interviewees. In Chile, stakeholder workshops were conducted at each of the three mine sites, with a total of 60 participants. Only six stakeholders attended one of the workshops due to social unrest that commenced in October. Seventeen key informant interviews were conducted. In Peru, 29 stakeholders took part in our workshop, and five key informant interviews were conducted.

**Table 1:** Participant characteristics

		**Sex**		**Stakeholder type**				
		**Female**	**Male**	**Mine staff**	**Community-based**	**Healthcare provider**	**Government official**	**Other**^a^
**Brazil**	**Workshop**	23	8	-	10	7	1	13
	**Interview**	7	6	4	0	3	2	4
**Chile**	**Workshop**	44	16	-	47	10	1	2
	**Interview**	12	5	6	5	3	1	2
**Peru**	**Workshop**	21	8	-	11	10	4	4
	**Interview**	1	4	1	2	0	2	0

^a^other stakeholders represented the education and social sectors, Guardianship council and religious groups in Brazil, community organisations in Chile and academic, media and business sectors in Peru

Table 2 shows the availability of quantitative data varied within and between countries, and across the SDG3 targets. In Brazil, a federal country, data availability was generally good at the state level, with local health authorities often producing epidemiological bulletins. Data availability became increasing sparse moving from state to municipality (see Table 2). In Chile, we retrieved data for all indicators at the national level. At the regional level, data availability was better within Valparaíso than within Metropolitana. At the district level within Metropolitana, only Lo Barnechea had some data availability. In Peru, data were available at only two levels of aggregation: national and regional. As such, data were sparse for the Mariscal Nieto province, and unavailable for Torata district.

**Table 2:** Data availability by area of interest and SDG3 target and indicator

		**SDG**									
		**3.1.1**	**3.1.2**	**3.2.1**	**3.3.1**	**3.3.2**	**3.3.3**	**3.3.4**	**3.3.5**	**3.4.1**	**3.4.2**
		**Maternal mortality ratio**	**Skilled birth attendance**	**Infant & child mortality**	**HIV**	**TB**	**Malaria**	**Hepatitis B**	**Neglected tropical diseases**	**Non-communicable diseases**	**Suicide**
**Brazil**											
**State**	**Minas Gerais**	✓		✓	✓	✓	✓	✓		✓	✓
** Municipality**	**Conceição do Mato Dentro**	✓		✓	✓	✓	✓	✓		✓	✓
**State**	**Goiás**	✓		✓	✓	✓	✓	✓		✓	✓
** Municipality**	**Barro Alto**	✓		✓	✓	✓	✓	✓		✓	✓
**Chile**											
**Region**	**Valparaiso**	✓	✓	✓	✓	✓				✓	✓
**Province**	**Quillota**	✓		✓	✓					✓	✓
**District**	**Nogales**	✓		✓						✓	✓
**Province**	**San Felipe de Aconcagua**	✓		✓	✓					✓	✓
**District**	**Catemu**	✓		✓						✓	✓
**Region**	**Metropolitana**	✓	✓		✓	✓				✓	✓
**Province**	**Santiago**										
**District**	**Lo Barnechea**					✓				✓	
**Province**	**Chacabuco**										
**District**	**Tiltil**										
**District**	**Colina**										
**Province**	**Andes**										
**District**	**Los Andes**										
**Peru**											
**Region**	**Moquequa**	✓	✓	✓	✓	✓	✓			✓	✓
**Province**	**Mariscal Nieto**										
**District**	**Torata**										

		**SDG**									
		**3.5.1**	**3.5.2**	**3.6.1**	**3.7.1**	**3.7.2**	**3.8.1**	**3.8.2**	**3.9.1**	**3.9.2**	**3.9.3**
		**Substance abuse disorder treatment**	**Alcohol consumption**	**Road traffic accidents**	**Contraception access**	**Adolescent birth rate**	**Universal health coverage**	**Health expenditure**	**Air pollution**	**Water, sanitation and hygiene for all**	**Unintentional poisonings**
**Brazil**											
**State**	**Minas Gerais**		✓	✓		✓		✓			
** Municipality**	**Conceição do Mato Dentro**		✓	✓		✓					
**State**	**Goiás**		✓	✓		✓					
** Municipality**	**Barro Alto**		✓	✓		✓					
**Chile**											
**Region**	**Valparaiso**		✓	✓		✓					
**Province**	**Quillota**			✓							
**District**	**Nogales**			✓							
**Province**	**San Felipe de Aconcagua**			✓							
**District**	**Catemu**			✓							
**Region**	**Metropolitana**		✓	✓		✓					
**Province**	**Santiago**										
**District**	**Lo Barnechea**			✓							
**Province**	**Chacabuco**										
**District**	**Tiltil**										
**District**	**Colina**										
**Province**	**Andes**										
**District**	**Los Andes**										
**Peru**											
**Region**	**Moquequa**		✓	✓	✓	✓	✓				
**Province**	**Mariscal Nieto**										
**District**	**Torata**										

### Brazil

In Brazil, stakeholders were consistent in reporting harmful substance use, access to sexual and reproductive health services, non-communicable diseases (NCDs) and communicable diseases as priority areas. There was also a good degree of agreement as to the individual, interpersonal, community, societal, and structural factors underlying these priority areas. At a structural level, there were calls to increase the number and capacity of health care providers and to better engage political leadership, and at a societal level to mobilise local advocacy and increase recreational facilities. An expressed need to address negative social and gender norms at the community level were linked to interpersonal level factors relating to gender inequalities, gender-based violence, “toxic masculinity” and “machismo”. Health needs were expressed in particular relation to young people (aged 13 to 29 years) in terms of harmful substance use and sexual and reproductive health.

**Fig. 2:**
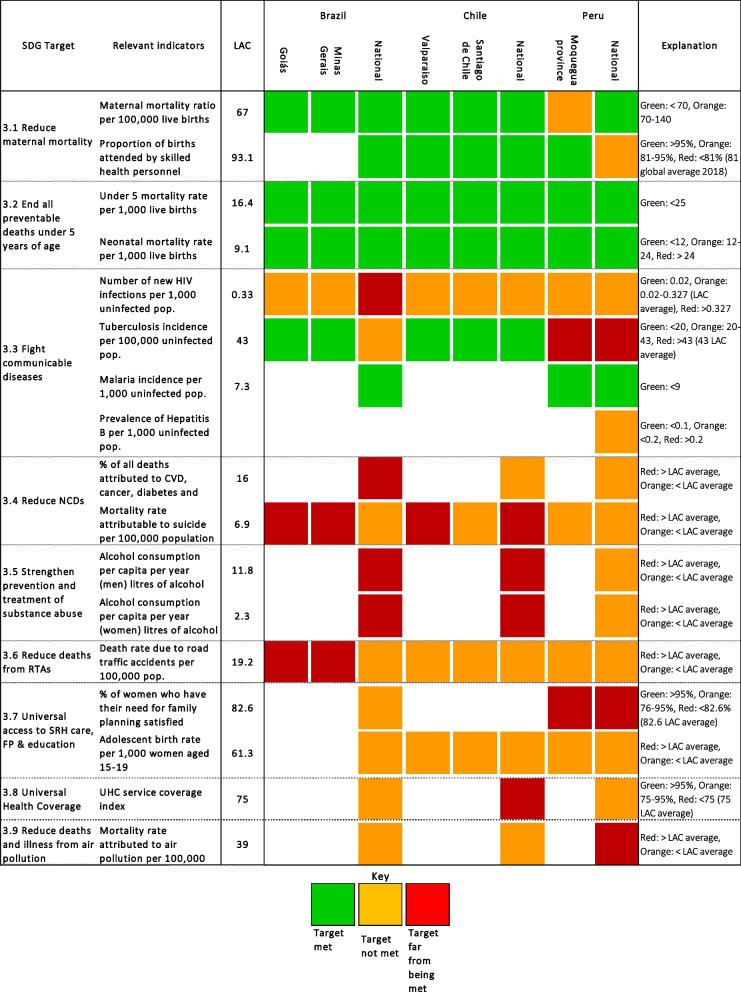
Heat map indicating progress against SDG3 indicators in Brazil, Chile and Peru

With the exception of the Human Immunodeficiency Virus (HIV), alcohol consumption, and deaths attributable to NCD, Brazil was consistently closer to the SDG3 indicator targets as compared to the LAC region as a whole (Fig. 2). Where data were available, all indicators, apart from HIV, suicide rates and road traffic accidents (RTAs), improved since 2000, with the targets for maternal and new-born mortality rates having been met. In host community states, the target for tuberculosis (TB) was met. Compared to the national average, the target for HIV was closer to being met whereas those for suicide and RTAs were further from being met.

### Chile

In Chile, stakeholders in Valparaíso region were consistent in prioritising action against NCDs, universal health coverage (UHC), and hazardous chemicals and pollution, whereas in the Metropolitan Region of Santiago de Chile, harmful substance use was the only target consistently reported, although sexual and reproductive health and communicable diseases were also raised frequently. A need to improve access to quality health care, and to tackle poverty and sub-optimal education as socio-economic determinants of ill-health, were identified as key structural and societal level factors, respectively. Stakeholders, particularly those in the Valparaíso region, expressed a need to better understand the impact of industrial and agricultural pollution on human health, and highlighted that tackling pollution was a prerequisite for any health and wellbeing intervention. Non-communicable diseases were discussed within the context of age. Among young people this priority was discussed in the context of poor self-esteem, and poor access to recreational or sports facilities; facilities which allow young people to engage in physical activity thereby potentially lessening issues relating to obesity and having too much “idle” time, an issue that was linked to substance use. Recreational engagement was also raised in the context of NCDs and an ageing population.

Chile’s progress towards the SDG targets was consistently better not only as compared to the LAC region average, but also as compared to Brazil and Peru. Exceptions to this were suicide, harmful alcohol consumption, and UHC. Compared to 2000, performance against the targets in Chile was good with the exception of HIV and alcohol consumption. Chile has already met a number of the SDG3 targets, including maternal, new-born and under-5 mortality, and TB incidence, and the proportion of births attended by a skilled health professional is high (Fig. 2).

### Peru

In Peru, stakeholders overwhelmingly prioritised action on two SDG3 targets: NCDs and hazardous chemicals and pollution. The need to address UHC was an underlying factor related to both priorities. A need to tackle structural level factors in terms of improving access to quality health care was expressed, as was a societal level need to tackle poverty, a key socio-economic determinant of ill-health. Although education per se was not raised as a determining factor locally, there was a consistent focus on the need to address environmental and health literacy and the mental health of young people. The topography of the local area (i.e. highland communities that can be difficult to access and have limited access to services), was raised in relation to structural, societal and community level factors impacting on health and wellbeing. More specifically, a reliance on subsistence husbandry, being reliant on the land for survival, was seen as a barrier to accessing health facilities due to the demands of the work and that such employment is rarely served by workplace health services.

At an interpersonal level, gender-based violence was a priority action area, whereas, at the individual level, gaps in knowledge leading to poor lifestyle choices and health seeking behaviour was a concern. A key finding in Peru relates to the socio-political tensions between the mining sector and host communities. A significant issue that emerged consistently from the qualitative data was the absence of evidence on the causes of pollution, the levels of air, water and soil pollution, and the impact of pollution on human health. Reportedly the lack of data exacerbates accusations of harmful practices, fuels a discourse that refutes or diminishes the problem, and has resulted in a lack of trust and constructive communications across all parties. There was a clear need to collect relevant data in a transparent manner to enable constructive engagement to improve strategies to mitigate impact of extractive practices.

Compared to the LAC region overall, and Brazil and Chile specifically (where data were available), Peru was performing less well against targets pertaining to TB, access to family planning, and mortality due to pollution. The opposite was true for alcohol consumption, RTAs, and NCD related mortality; in particular mortality attributable to cardiovascular diseases. In particular, little progress at the national and regional level has been made against mortality from air pollution and access to modern contraception (Fig. 2).

### Qualitative and quantitative assessment alignment

The quantitative assessments in each of the three countries generally aligned with the priorities identified in the qualitative data. Although insufficient data availability did not allow a full assessment of the extent of alignment across all indicators, clear patterns emerged. Qualitative and quantitative data consistently identified NCDs (including mental health), harmful substance use (particularly in terms of harmful alcohol consumption) and pollution (particularly air pollution) as areas for priority action (see Table 3). The NCD burden of mortality across the countries was comparable in each of the four major disease groups (cardiovascular disease, cancer, diabetes and respiratory diseases). The qualitative and quantitative data that were available also suggested targets relating to TB and adolescent birth rates in Brazil and Chile, and UHC in Chile and Peru, required particular attention. We should note that in Peru, TB was identified as a clear priority area for action nationally, but this was less clear from quantitative data at the local level and was not raised as a priority in the qualitative data (potentially due to TB being less prevalent in the high-altitude regions). When interpreting these findings, it should be noted that often an absence of quantitative data below the national and province level limited data triangulation in the three countries.

**Table 3:**
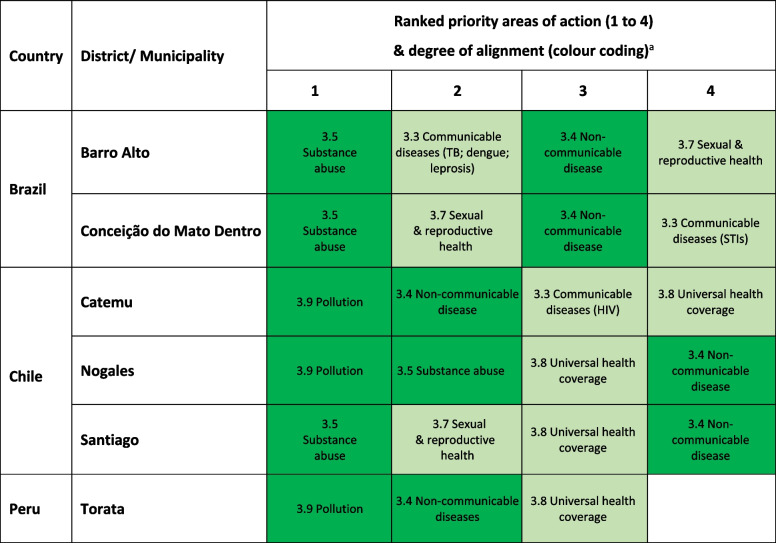
Health priorities ranking by site according to the qualitative findings, and extent of alignment with the quantitative data

Colour coding for alignment:

^a^Two-step results process in which health priorities were ranked in order of importance during the qualitative appraisal and then compared against the quantitative data to assess the extent of alignment

## Discussion

We present a mixed-method approach that demonstrates the importance of engaging with local populations, as well as local and national stakeholders, to contextualise the health priorities of mining communities in Brazil, Chile and Peru, in line with the SDG3 targets. We also highlight how an independent academic consortium can support public private partnerships and private sector investment to focus on priority areas to advance the SDG agenda.

Where data were publicly available, we found good alignment between the quantitative data and priority areas identified qualitatively by stakeholders, and between what was reported at the national and state/region and district levels. We also highlight important individual, interpersonal, community, societal, and structural factors underlying these priority areas that require action in order to make progress to the SDG3 targets. As compared to the LAC region, our countries of interest generally performed well against the SDG3 targets. There were exceptions though, with Brazil at the national level doing less well in relation to HIV, alcohol consumption, and NCD-related deaths, Chile doing less well in relation to suicide, harmful alcohol consumption, and UHC, and Peru under-performing against TB, family planning, and pollution-related deaths.

Across the three countries, NCDs (SDG3.4), harmful alcohol consumption (SDG3.5), and pollution, particularly air pollution (SDG3.9), were consistently identified as areas for priority action. In relation to NCDs, there was comparable burden of mortality across the countries in each of the four major diseases groups (cardiovascular disease, cancer, diabetes and respiratory diseases) and suicide rates. In addition to sharing a common mortality burden, key drivers for NCDs were also similar across the three countries. These factors included mental health issues, harmful substance use, and inequality in access to essential health-care services. Increased incidence of NCDs is also driven by broad epidemiological transitions in the region, the most important being increased life expectancy [13].

Although the qualitative data identified air pollution as a priority area for action, a paucity of available quantitative data meant we could not quantify its health impact. In both Chile and Peru, a need to better understand the impact of pollution on human health, and the role of mining in this, was expressed. In Peru, it was suggested a lack of data exacerbated the discourse about the impact of mining practices and resulted in a lack of trust and constructive communication. Reductions in health-related quality of life, increased perceptions of detrimental health conditions, and higher frequency of medical consultations have been reported in populations living close to mining settlements [14–16]. However, health conditions associated with mining operations have multifactorial aetiology with biological and social risk factors (behaviours, lifestyle and living conditions) that are complex and often difficult to quantify and disentangle [17]. Robust data on the impacts of mining operations on health outcomes could inform interventions to reduce health risks. To this end, there is an opportunity for mining companies, and appropriate partners, to adopt robust methodologies to conduct environmental impact assessments and generate an evidence base to inform interventions.

Other areas identified as requiring particular attention were TB (SDG3.3) and adolescent birth rates (SDG3.7) in Brazil and Chile, and UHC (SDG3.8) in Chile and Peru. An assessment of only the quantitative data in Peru would have led us to assume TB was a priority at the local level, however, the qualitative data highlighted that this was not the case.

Our study has several limitations. As our criteria for community inclusion was distance from a mining operation, we did not consider potential demographic differences between communities. It is possible that differences identified in local health needs may reflect population level differences between communities, as well as differences between and within countries in relation to national or provincial public health policy priorities.

An important limitation is that due to different reporting systems and differing public availability of data (some data could only be accessed via formal data access requests) within and between the three countries, quantitative data were often scarce at the district level, and unavailable at the level of host community. Where there is a paucity of data, there is a need for careful consideration when interpreting our findings. Our mixed-methods approach helped reduce the impact of limited data by facilitating the triangulation of data (i.e. where quantitative data were not available at a local level, qualitative findings helped fill the data gaps). Reflecting the challenges of measuring against rigid global indicators, there was relatively little data publicly available on outcomes not directly related to mortality within the health system.

The authors of a recent evaluation of SDGs aimed at local communities in LAC also reported that there was a scarce availability of standardised, open, and comparable data [18]. The COVID-19 pandemic not only clearly demonstrated the need for countries to strengthen their health data and information systems, but also how required robust data systems could be implemented when necessary resources were made available [19]. If concerns relating to sustainability are addressed [20], such systems may present opportunities to address wider deficiencies in the availability of quantitative data.

To address the health priorities identified in each country, intervention packages tailored to the specific needs of host communities, and integrated within local and national systems, structures and partnerships, are required. To facilitate this, we need to gather robust data at the local level to confirm the actual burden and determinants of those health issues indicated, and to direct and track progress. Appropriate monitoring systems should be implemented, as should epidemiological investigations into the local context, and rapid assessments of health risks. In a recent study of SDG3-related inequalities in the LAC region, the authors recommended the collection of disaggregated data on the health and well-being of women, children and adolescents, to allow for evidence-based planning [21]. The authors also advocated for further exploration of the drivers and mechanisms underlying the inequalities observed.

For interventions to successfully and sustainably address the local health priorities identified, it is essential they tackle associated upstream factors, including poor access to quality healthcare and medication, as well as societal level factors, including socio-economic determinants of ill-health such as poverty and insufficient education. At a community level there is a need to tackle gaps in knowledge that undermine health seeking behaviour and, specific to this context, there is a need to implement transparent processes in order to build trust between the mining sector and host communities. Furthermore, future interventions are more likely to be successful if sustained by participatory approaches, taking into account the voice and perspectives of host communities.

Although Brazil, Chile and Peru have made progress in developing social protection platforms and UHC coverage systems, access to quality health services remains inequitable. Successful interventions to tackle the identified health priorities are likely to sit at the intersection of these two programmatic actions. As such, close collaborations between governmental and non-governmental partners are required to better integrate social services and social protection with health and strengthen financial and political commitments to improve health infrastructure and address structural drivers.

## Conclusions

In the host communities in Brazil, Chile and Peru we studied, there is a clear imperative to address SDG3 priority targets. In achieving this, intervention packages tailored to the specific needs of host communities, integrated within local and national systems, structures and partnerships, and that tackle associated upstream factors and help further social protection platforms and UHC coverage systems, are required. There is the potential for the mining sector to be a key health partner in supporting collaborative regional development, including promoting integrated programmes and strengthening health systems. Investment from private industry more broadly in health financing, promoting public private partnerships, and addressing the harmful impacts of their activities forms a crucial part of implementing programmatic activities for achieving the SDG3 health goals.

## Data Availability

The datasets used and/or analysed during the current study available from the corresponding author on reasonable request.

## Abbreviations

SDG: Sustainable Development Goals
RTA: Road Traffic Accidents
NCD: Non-Communicable Disease
UHC: Universal Health Coverage
MMR: Maternal Mortality Ratio
TB: Tuberculosis
HIV: Human Immunodeficiency Virus
LAC: Latin America and Caribbean
